# Robotic Guided Knee Arthroplasty - Group Learning Curve and Early Outcomes

**DOI:** 10.1016/j.artd.2025.101746

**Published:** 2025-07-02

**Authors:** Max Gordon, Gurion Rivkin, Alex Greenberg, Leonid Kandel, Meir Liebergall, Itay Perets

**Affiliations:** Orthopedic surgery department, Hadassah Medical Organization, Hadassah University Hospital, Israel, Jerusalem

**Keywords:** Robotic assisted surgery, Knee arthroplasty, Learning curve

## Abstract

**Background:**

Previous studies have reported initial and proficiency phases of the learning curve among individual surgeons when robotic-assisted total knee arthroplasty (raTKA) into their practices. The purpose of this study was to assess the number of raTKA cases needed to achieve proficiency in the operative time (OT) across a group of surgeons.

**Methods:**

A retrospective analysis comparing raTKA to manual Total Knee Arthroplasty (mTKA) was performed at a single-site comprised of 6 orthopaedic surgeons. The first 90 patients who underwent raTKA at the institution divided chronologically into 3 groups of 30 each and compared to 30 previous mTKA cases. Tourniquet time was used as the measure for OT, range of motion (ROM) was measured preoperatively and at 2 and 6 weeks postoperative.

**Results:**

Average OT was significantly shorter in the mTKA group in comparison with all the raTKA groups. A statistically significant difference was found in OT when comparing the succeeding raTKA groups (mean time of 97.6, 86.6, and 76.7 min *P* value<.001). The change in ROM at 6 weeks postoperative was found to be greater in the first raTKA group in comparison to the second raTKA group (mean of 0.9° vs −17.1°; *P* = .04) and the mTKA group (mean of 0.9° vs −7.9°; *P* = .04). However, when controlling for preoperative ROM and performing surgeon, there was no difference in ROM at 6 weeks postoperative between defined time periods (*P* = .78). No difference was found when comparing rate of complications (*P* value>.05).

**Conclusions:**

When evaluating the group learning curve, this study showed average shorter operating time with every 30 cases (mean time of 97.6; 86.6- and 76.7-min *P* value<.001) and no difference in complication rate (*P* value>.05). These findings suggest continued proficiency over time; however, adoption of the technology is associated with longer OTs.

## Introduction

Total knee arthroplasty (TKA) is considered one of the most successful surgeries providing better functionality and quality of life in most patients with a 10-year failure rate of approximately 5% [1]. A variety of surgical philosophies exist regarding intraoperative variables such as type and alignment of implants, soft tissue release/preservation, ligament tensioning, and the degree of flexion-extension gap balancing. While each of these variables may influence the function and stability of the knee [2] and the longevity of the implant [3,4], malalignment of the implant is the most common documented etiology leading to aseptic failure [5]. New technologies allow for more precise component placement [6,7], providing surgeons with greater control over intraoperative factors [8], and may further improve clinical outcomes following TKA [9].

Robotic-assisted TKA (raTKA) is increasing in popularity and several systems have already shown improved accuracy compared to nonrobotic TKA using manual Total Knee Arthroplasty (mTKA) with recent studies suggesting improved early patient-reported outcomes [[9], [10], [11], [12], [13]]. When evaluating the integration of these systems, several studies have described the complexity of adoption while accounting for many variables such as learning curve [8,14], workflow [15], accuracy [16] and clinical outcomes [9,17].

The robotic systems available commercially vary in design and a relatively newer model uses a surgical arm to place and hold cutting jigs for bone resection. This method allows the surgeon to remain in control of the saw while attaining robotically accurate bone resections [15]. Furthermore, valuable data regarding soft tissue balance are provided in real-time, enhancing intraoperative surgical decision-making. This system has been shown to have high accuracy in component placement in a single-arm study [[18], [19], [20]], along with improved component placement compared to mTKA [21,22]. However, novel technologies must be evaluated in aspects beyond accuracy including their effect on the operative time (OT), complication rate, and clinical outcomes.

Recent literature has suggested initial learning curves ranging from 5 to 15 cases with no apparent outcome changes specific to this system [14,23]. Additionally, Kenanidis et al. [24] have proposed that roughly 70 cases are needed to become proficient with the system regarding achieving similar surgical times to mTKA. These studies have focused on both the initial and proficiency phases of the learning curve among individual surgeons when integrating this system into their practices. However, we are unaware of any previous studies demonstrating the effect of adoption across a group of surgeons.

As such, we sought to assess the number of raTKA cases needed to achieve proficiency in the OT across the group and to evaluate range of motion (ROM), comparing these to standard mTKA. Additionally, we compared postoperative hospitalization times and complications.

## Material and methods

After obtaining approval from an ethics committee, we conducted a retrospective, single-site, cohort study on a series of raTKA and mTKA cases performed by a trained arthroplasty team. Six, high-volume, arthroplasty specialist, orthopaedic surgeons contributed cases to this review. Starting January 2020 until early 2022, we collected data from 30 mTKA patients and the first 90 consecutive raTKA cases in patients over the age of 18 undergoing primary TKA for treatment of end-stage osteoarthritis. No cases of traumatic or rheumatoid arthritis were included in this analysis. Patient demographic data were collected including age, body mass index, and sex (Table 1).

**Table 1:** Patient characteristics for all 4 groups.

Characteristic	raTKA-1	raTKA-2	raTKA-3	mTKA	*P* value
Age, mean years	68.7	69.5	71.9	70.2	.27
Sex, male, n (%)	12 (40%)	14 (46%)	14 (46%)	8 (26%)	.92
BMI, mean	30.7	29.4	30.6	30.1	.6
Lateralization-right, n (%)	18 (60%)	11 (36%)	16 (53%)	15 (50%)	.47
Implant (N-Nexgen/P-Persona), n	N-13/P-17	N-17/P-13	N-12/P-19	N-23/P-7	**.018**
Hospitalization days, n	3.2	2.7	2.2	3.1	**.02**

BMI, body mass index (kg/m^2^).

Bold values indicate statistical significance.

All surgeries were performed according to the ROSA Knee System’s (Zimmer Biomet, Montreal, Quebec, Canada) surgical technique. All patients received either the Persona or NexGen Knee System (Zimmer Biomet, Warsaw, IN, USA). The prevalence of each implant system per group showed a statistically significant difference between mTKA and the first and third raTKA groups (Table 2). All patients followed the same postoperative care pathway, including follow-up at 2 and 6 weeks, as well as 1 year postoperative.

**Table 2:** Comparison of implant types between the subgroups.

Implant type	raTKA-1	raTKA-2	raTKA-3	mTKA	ꭓ2 *P* value
Implant (N-Nexgen/P-Persona), N	N-13/P-17^a^	N-17/P-13	N-12/P-19^a^	N-23/P-17	.018

a Significantly different than mTKA, *P* < .01.

Tourniquet time was used as the measure for OT. Tourniquet protocol is the same with all arthroplasty surgeons—it is inflated right before making the initial incision and is deflated after applying the sterile dressing. Additionally, surgical data regarding the side of surgery, OT, and type of implant were recorded. Patient’s knee ROM on the operated side was measured and documented preoperatively, and at 2 weeks and 6 weeks follow-up. ROM was calculated by deducting maximum extension angle from maximum flexion angle resulting in a final “arc of motion.” When comparing the change in ROM at different time points, the preoperative arc of motion was deducted from the two- and 6-week postoperative arc of motion.

Postoperative duration of hospitalization days was calculated. Follow-up period for complications was 1 year and included intraoperative and postoperative complications. Complication rates were further divided into periods of up to 3 months and 1 year.

Single-surgeon-oriented studies reported a learning curve of 6-40 cases [23,[25], [26], [27]], thus the first 90 patients who underwent raTKA at our institution were used and were divided chronologically into 3 groups of 30 each. These groups were compared to 30 mTKA cases performed immediately prior. This convenience sample was considered sufficient to produce a group learning curve for the raTKA cohort. Based on previous work [28,29], we presumed minimal clinically important difference for ROM as 10 degrees and a standard deviation of -\+ 10. Thus, to achieve an alpha of 0.05 and power of 90%, the a priori power analysis demonstrated the need of 22 patients in each group which is covered in the convenience sample for the learning curve. Continuous variables are presented as means and standard deviations. Comparison of 2 continuous variables was made using independent *t*-tests. Comparison of mean ROM at 6 weeks postoperative, considering preoperative ROM, surgeon, and time period, was done using an analysis of covariance. Categorical data were counted and comparison of these variables was analyzed with a chi-square test. Surgical duration was evaluated and a logarithmic trendline curve was computed. Statistical significance was set at *P* < .05 for all statistical tests.

## Results

There were no differences in age, sex, or body mass index between the groups (*P* > .05, Table 1). Distribution of raTKA cases between the surgeons is depicted in Table 3.

**Table 3:** Distribution of raTKA cases between the surgeons.

Surgeon	raTKA-1	raTKA-2	raTKA-3	Sum
1	-	10	1	11
2	-	1	7	8
3	12	10	4	26
4	7	1	4	12
5	5	2	1	8
6	6	6	13	25
Total, n	30	30	30	90

**Figure 1:**
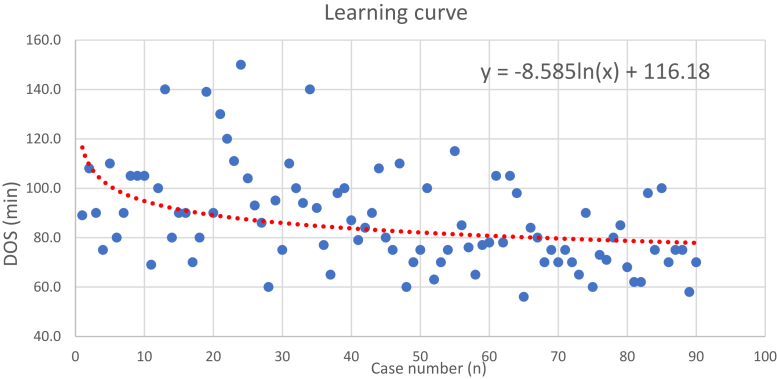
Learning curve of first 90 cases raTKA. The learning curve is represented by the exponential trendline and its equation in the top right corner of the figure.

Average OT was significantly shorter in the mTKA group in comparison with all the raTKA groups. A statistically significant difference was found in OT when comparing the succeeding raTKA groups (Table 4). When comparing OT in 3 raTKA between the 6 surgeons, no statistically significant difference was found in any of the comparisons (*P* > .05). When comparing the 2 most prominent surgeons with the other surgeon, still, no difference was found (*P* > .05) (Table 5). The learning curve that was created from the first 90 raTKA is described by the following equation—y = −8.585ln(x) + 116.18 (Fig. 1). The data suggest continued proficiency over time.

**Table 4:** Operative time comparison between the different groups.

	raTKA-1	raTKA-2	raTKA-3	mTKA	ANOVA *P* value
OT, minutes (mean)	97.6^a^^b^	86.6^b^	76.7^a^^b^^c^	53.7	<.001

a Significantly different than raTKA-2, *P* < .05.

b Significantly different than mTKA, *P* < .0001.

c Significantly different than raTKA-1, *P* < .0001.

**Table 5:** Operating time—prominent surgeons vs rest of surgeons.

Surgeon	raTKA group	Mean	Standard deviation	*P* value
3 + 6	raTKA-1	92.7	18.1	.16
1 + 2 + 4 + 5 + 6	raTKA-1	105	25.6	
3 + 6	raTKA-2	81.1	18.7	.07
1 + 2 + 4 + 5 + 6	raTKA-2	92.7	16	
3 + 6	raTKA-3	73.5	110.5	.1
1 + 2 + 4 + 5 + 6	raTKA-3	81	16.2

The preoperative average arc of motion found in the first, second, third raTKA, and mTKA group were 100.6, 107.4, 99.8, and 108.3, respectively. There were no significant (*P* > .05) differences in the initial ROM. Similarly, there were no significant (*P* > .05) differences in ROM change at 2 weeks postoperative between groups. The change in ROM at 6 weeks postoperative was found to be greater in the first raTKA group in comparison to the second raTKA group (mean of 0.9° vs −17.1°; *P* = .04) and the mTKA group (mean of 0.9° vs −7.9°; *P* = .04) (Table 6). Analysis of covariance results suggest that after considering preoperative ROM and performing surgeon, there was no difference in ROM at 6 weeks postoperative between defined time periods (*P* = .78).

**Table 6:** Postoperative change in ROM arc.

Measurement	raTKA-1	raTKA-2	raTKA-3	mTKA	ANOVA *P* value
Preop ROM (degrees), mean (SD)	100.6 (16.4)	107.4 (13.7)	99.8 (13.6)	108.3 (15.6)	.052
2-week postop ROM (degrees), mean (SD)	−25.6 (23.1)	−34.9 (36.4)	−26.6 (23.7)	37.2 (37.2)	.26
6-week postop ROM (degrees), mean (SD)	0.9 (15.3)	−17.1 (46)^a^	0.5 (17)	−7.9 (18.2)^a^	**.03**

Bold values indicate statistical significance.

a Significantly different than raTKA1, *P* < .05.

Duration of postoperative hospitalization was shorter (*P* < .05) in the third raTKA group by approximately 1 day when compared to the first raTKA and the mTKA groups (Table 7).

**Table 7:** Comparison of hospitalization period between the different groups.

	raTKA-1	raTKA-2	raTKA-3	mTKA	ANOVA *P* value
Hospitalization days, n	3.2	2.7	2.2^a^^b^	3.1	**.02**

Bold values indicate statistical significance.

a Significantly different than raTKA1, *P* < .001.

b Significantly different than mTKA, *P* < .05.

When comparing complication rate between the 4 groups, there were no significant differences for specific complications (revision, coagulopathy events, manipulation rate) nor for total complications rate (Table 8).

**Table 8:** Complication rate.

Complication	raTKA-1	raTKA-2	raTKA-3	mTKA	*P* value
Revision	0	0	1	0	.38
Manipulation	0	2	1	3	.31
Coagulopathies	0	0	1	0	.38
Total	0	2	3	3	.35

## Discussion

The main finding of this study was that proficiency in this system continues to develop beyond 60–90 raTKA cases (Fig. 1) in a group of 6 orthopaedic surgeons. We found that the mean OT was shortened by about 10 minutes between the first and second raTKA groups. OT was shortened by a similar time between the second and third groups. Additionally, our data suggest better ROM at 6 weeks postoperative, favoring the first raTKA. There was also a roughly 1 day decrease in hospitalization in the final raTKA group compared to both the first raTKA group and the mTKA group. This was statistically significant and may indicate value in reducing hospitalization following TKA. There was no difference in complication rates.

Learning curves have been measured for the use of raTKA by general orthopaedic surgeons and orthopaedic surgeons specializing in arthroplasty [30]. Using a logarithmic trendline of the OT from all 90 raTKA, we produced a group learning curve. The learning curve represents the groups’ confidence with the robotic system and its incorporation into the workflow. Various studies have reported differing learning curves for raTKA, ranging from 6 to 43 cases [25,27,[31], [32], [33]]. A recent retrospective study using the ROSA system reported a learning curve of 6-11 cases [23]. However, they did not report on the proficiency phase of the learning curve. Interestingly, in a single-center study on the learning curve of 3 surgeons, Bolam et al. [14] reported an inflexion point of 5-15 cases with no difference in OTs between robotic and manual TKA. More recently, Kenanidis et al. [24] suggested that OTs were balanced at roughly 70 raTKA cases compared to manual TKA using the ROSA Knee System. When evaluating another system, Marchand et al. have reported reaching OT that is similar to mTKA after 20-40 cases of a single surgeon [30]. The studies hereby mentioned are all single-surgeon oriented. In our study, we follow cases from 6 different surgeons and a heterogeneous operating room team. This evaluation allows more accurate portrayal of the incorporation of the ROSA Knee System into the workflow in our center.

The learning curve presented in our study describes a starting OT of almost 2 hours for a surgeon not familiar with raTKA. Our results demonstrate an ongoing improvement in OT within the first 90 cases. Within these 90 cases, we managed to produce a group learning curve that has yet presented a plateau that would represent a stabilization in surgery time. Our data are comparable to the previous data shown, when considering diversified operating team of 6 surgeons compared to the single-surgeon orientation. With this trend, further proficiency in the system is likely to be found across our practice and the speed of adoption by each surgeon will likely dictate this. As mentioned above, numerous studies have shown operating time for raTKA can be similar to mTKA after sufficient practice [30,34,35]. However, our study emphasizes that the implementation time of a new robotic surgical tool might be longer in a heterogenic team, rather than a single-surgeon-oriented training.

As suggested by Hopper et al., learning curve evaluations must include safety and outcome measurements [36]. Prior studies including one meta-analysis from 2019 showed no difference in ROM in a follow-up period of up to 2 years when comparing raTKA and mTKA [[37], [38], [39]]. Our study found a statistically significant difference, in the assessment of arc of motion at 6 weeks postoperative, between the first raTKA and the third raTKA and mTKA groups (Table 6). A possible explanation to this may lay in the relatively high preoperative arc of motion in these groups. It could be suggested that patients with an initial big arc of motion take longer to reach similar ranges and therefore, in the form of presentation hereby mentioned, appear to have lost more of their ROM. This difference was not found when assessing the change in arc of motion in the other raTKA and mTKA groups, nor when controlling for surgeon or preoperative ROM. These findings are consistent with the recent report by Fary et al. [40] who suggested earlier gains in active ROM using this robotic system compared to conventional. Furthermore, Mancino et al. [17] have recently noted improved ROM at 12 months following raTKA compared to navigated TKA using this robotic system.

Cost-effectiveness must be evaluated in the process of adopting a new technology, in which the postoperative hospitalization period should be considered. Held et al. have suggested no difference in the length of stay when comparing raTKA and mTKA [37]. However, Kayani et al. presented data showing reduced pain, better early functional recovery, and reduced hospital stay by 1 day on average for patients after raTKA [41]. Our data showed shortened hospitalization period in the final raTKA group that was significantly shorter by approximately 1 day when compared to the mTKA and first raTKA groups (Table 8). This difference can be attributed to the familiarity with the postoperative management and clinical follow-up of the patient after raTKA and is similar in length to other studies reporting shortened length of stay with raTKA [42,43].

Another goal of the study was to evaluate the safety of raTKA with this robotic system by comparing complication rates between the raTKA and mTKA groups. This topic has been addressed repeatedly in the literature in recent years, with conflicting results. Zhang et al. reported in a meta-analysis no difference when comparing wound complications from raTKA compared to mTKA [9], whereas Ofa et al. presented more prevalent revisions and manipulations with mTKA in a nationwide database study [44]. Some studies have shown lower rates of blood loss [39,41,45], coagulopathies [46] and other complications with raTKA. A recent study even tested whether raTKA is associated with decreased odds of early revision when compared to mTKA—not proving a difference [47]. In contrast, Wang et al. presented increased risk for surgical site infection and anemia with raTKA [48]. In the present study, there were no differences in the postoperative complication rate between raTKA and mTKA.

Our study has several limitations, including those inherent to a retrospective cohort study design. However, as data were prospectively collected and evaluated on consecutive cases, the risk of recall bias and selection bias was reduced. Second, this study describes surgeries of multiple surgeons to better understand the incorporation of the robotic system into the workflow. Nevertheless, the number of surgeries performed by each of the 6 surgeons is not equal. This could cause a bias in the learning curve and may explain the continued proficiency described in our data. However, the evaluation of this group learning curve presents better ecological validity regarding the adoption of raTKA in a hospital system. It is also worth mentioning that this study examined the surgeons’ effect on the workflow and results the surgery. Yet, it important to acknowledge that surgical technicians, OR nurses, and residents also plat a significant part in shortening OT and improved proficiency. Our institution works with a homogenous team assigned to arthroplasty cases. However, the staff may still differ from case to case. This study did not evaluate the effect of the total personnel aforementioned. An additional weakness of our study regard the implant types used. While a statistically significant difference in implant types was not found among the different raTKA groups, when comparing raTKA to mTKA, the latter group was found to have more NexGen implants than the raTKA group. This matter had no impact on the learning curve as the mTKA is the control group. Yet, it can still influence other parts of the analysis, though in our institute both implants are regularly used.

## Conclusions

When evaluating the group learning curve, this study showed average shorter operating time with every 30 cases (mean time of 97.6, 86.6- and 76.7-min *P* value<.001) and no difference in complication rate (*P* value>.05). These findings suggest continued proficiency over time; however, adoption of the technology is associated with longer OTs.

## Conflicts of interest

M. Liebergall serves as a Zimmer Biomet consultant for research and teaching without compensation and receives research support from 10.13039/100012630Zimmer Biomet as a Principal Investigator. L. Kandel is an unpaid consultant for 10.13039/100012630Zimmer Biomet and has Medical/Orthopaedic publications editorial/governing board from CORR. G. Rivkin received research support from Zimmer as a Principal Investigator; all other authors declare no potential conflicts of interest.

For full disclosure statements refer to https://doi.org/10.1016/j.artd.2025.101746.

## CRediT authorship contribution statement

**Max Gordon:** Writing – original draft, Investigation, Formal analysis, Data curation. **Gurion Rivkin:** Supervision, Formal analysis, Data curation. **Alex Greenberg:** Resources, Formal analysis, Data curation. **Leonid Kandel:** Supervision, Data curation, Conceptualization. **Meir Liebergall:** Supervision, Resources, Project administration, Methodology, Data curation, Conceptualization. **Itay Perets:** Writing – review & editing, Writing – original draft, Supervision, Project administration, Methodology, Formal analysis, Data curation, Conceptualization.

## Figures and Tables

**Conflict of Interest Statement for Rivkin:**
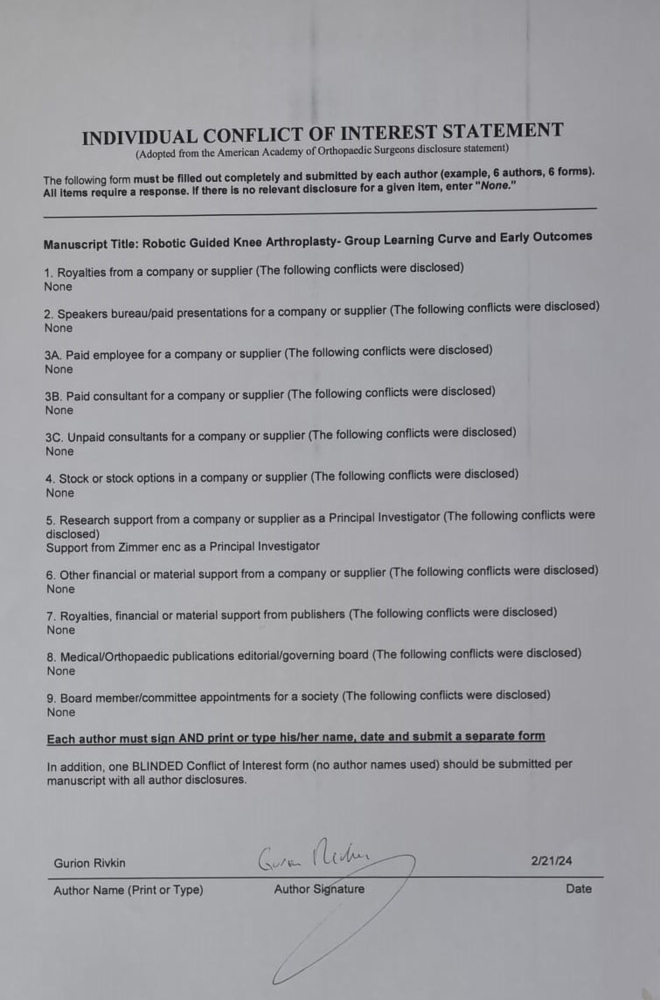


**Conflict of Interest Statement for Perets:**
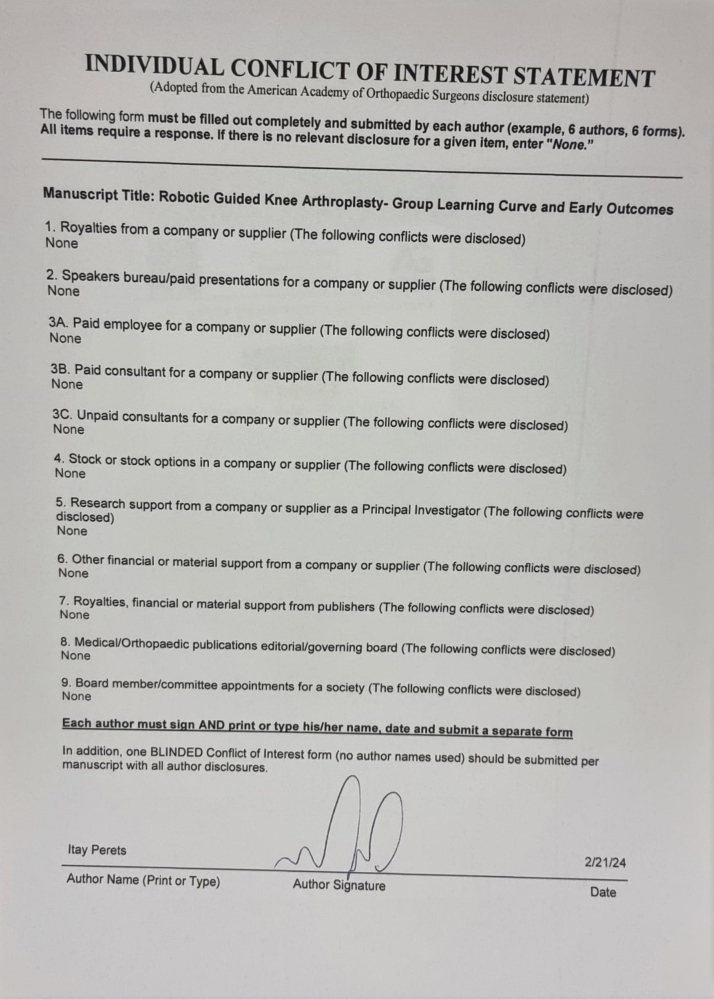


## Appendix A Supplementary data

Conflict of Interest Statement for Liebergall

Conflict of Interest Statement for Greenberg

Conflict of Interest Statement for Kandel

Conflict of Interest Statement for Gordon
