# Supplementary material for: Robotic Guided Knee Arthroplasty - Group Learning Curve and Early Outcomes
Source: Arthroplast Today. 2025 Jul 2;34:101746. doi: 10.1016/j.artd.2025.101746 (PMC12269883; doi:10.1016/j.artd.2025.101746)
Supplement: Conflict of Interest Statement for Greenberg [file mmc2.pdf]

INDIVIDUAL CONFLICT OF INTEREST STATEMENT

(Adopted from the American Academy of Orthopaedic Surgeons disclosure statement)

The following form **must be filled out completely and submitted by each author (example, 6 authors, 6 forms). All items require a response. If there is no relevant disclosure for a given item, enter "*None.*"**

Manuscript Title: Robotic Guided Knee Arthroplasty- Group Learning Curve and Early Outcomes

1. Royalties from a company or supplier (The following conflicts were disclosed)
None

2. Speakers bureau/paid presentations for a company or supplier (The following conflicts were disclosed)
None

3A. Paid employee for a company or supplier (The following conflicts were disclosed)
None

3B. Paid consultant for a company or supplier (The following conflicts were disclosed)
None

3C. Unpaid consultants for a company or supplier (The following conflicts were disclosed)
None

4. Stock or stock options in a company or supplier (The following conflicts were disclosed)
None

5. Research support from a company or supplier as a Principal Investigator (The following conflicts were disclosed)
Serves as Zimmer Biomet consultant for research and teaching without compensation
Research support from Zimmer Biomet as a Principal Investigator

6. Other financial or material support from a company or supplier (The following conflicts were disclosed)
None

7. Royalties, financial or material support from publishers (The following conflicts were disclosed)
None

8. Medical/Orthopaedic publications editorial/governing board (The following conflicts were disclosed)
None

9. Board member/committee appointments for a society (The following conflicts were disclosed)
None

<u>Each author must sign AND print or type his/her name, date and submit a separate form</u>

In addition, one BLINDED Conflict of Interest form (no author names used) should be submitted per manuscript with all author disclosures.

Prof. Meir Liebergall
Chairman Orthopedic Surgery
Hadassah Medical Organization

Meir Liebergall

2/26/2024

Author Name (Print or Type) | Author Signature | Date

Author Name (Print or Type)	Author Signature	Date
